# Ethyl 6-(4-ethoxy­phen­yl)-4-(furan-2-yl)-2-oxocyclo­hex-3-ene-1-carboxyl­ate

**DOI:** 10.1107/S1600536808040130

**Published:** 2008-12-10

**Authors:** Amir Badshah, Aurangzeb Hasan, Cecilia R. Barbarín, Asghar Abbas, Sajid Ali

**Affiliations:** a Department of Chemistry, Quaid-i-Azam University, Islamabad 45320, Pakistan; b División de Estudios de Posgrado, Facultad de Ciencias Químicas, UANL, Guerreo y Progreso S/N, Col. Treviño, CP, 64570, Monterrey, NL, Mexico

## Abstract

The title compound, C_21_H_22_O_5_, was prepared by NaOH-catalysed cyclo­condensation of 3-(4-ethoxy­phen­yl)-1-(furan-2-yl)prop-2-en-1-one with ethyl acetoacetate. In the crystal, C—H⋯O and C—H⋯π inter­actions link the mol­ecules. In the title mol­ecule, the furan and cyclo­hexene rings are almost parallel [6.77 (11)°] and the cyclo­hexene ring is approximately perpendicular to the benzene ring [84.79 (5)°].

## Related literature

For background to cyclo­hexenones, see: Eddington *et al.* (2000 ▶); Li & Strobel (2001 ▶); Luu *et al.* (2000 ▶); Padmavathi *et al.* (2000 ▶, 2001 ▶).
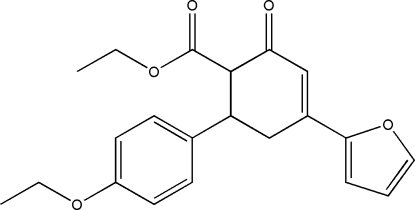


## Experimental

### 

#### Crystal data

C_21_H_22_O_5_
*M* *_r_* = 354.39
Monoclinic, 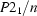

*a* = 7.361 (3) Å
*b* = 17.350 (4) Å
*c* = 14.473 (3) Å
β = 104.07 (2)°
*V* = 1792.8 (9) Å^3^
*Z* = 4
Mo *K*α radiation
μ = 0.09 mm^−1^
*T* = 298 (2) K
0.60 × 0.40 × 0.40 mm

#### Data collection

Bruker P4 diffractometer
Absorption correction: none
8745 measured reflections
5218 independent reflections
3806 reflections with *I* > 2σ(*I*)
*R*_int_ = 0.021
3 standard reflections every 97 reflections intensity decay: 3.4%

#### Refinement

*R*[*F* ^2^ > 2σ(*F* ^2^)] = 0.047
*wR*(*F* ^2^) = 0.135
*S* = 1.06
5218 reflections
237 parameters
H-atom parameters constrained
Δρ_max_ = 0.27 e Å^−3^
Δρ_min_ = −0.17 e Å^−3^

### 

Data collection: *XSCANS* (Siemens, 1999 ▶); cell refinement: *XSCANS*; data reduction: *XSCANS*; program(s) used to solve structure: *SHELXTL-Plus* (Sheldrick, 2008 ▶); program(s) used to refine structure: *SHELXTL-Plus*; molecular graphics: *SHELXTL-Plus* and *Mercury* (Macrae *et al.*, 2006 ▶); software used to prepare material for publication: *SHELXTL-Plus*.

## Supplementary Material

Crystal structure: contains datablocks I, global. DOI: 10.1107/S1600536808040130/fb2116sup1.cif

Structure factors: contains datablocks I. DOI: 10.1107/S1600536808040130/fb2116Isup2.hkl

Additional supplementary materials:  crystallographic information; 3D view; checkCIF report

## Figures and Tables

**Table 1:** Hydrogen-bond geometry (Å, °)

*D*—H⋯*A*	*D*—H	H⋯*A*	*D*⋯*A*	*D*—H⋯*A*
C3—H3*A*⋯O3^i^	0.93	2.55	3.441 (2)	160
C19—H19*A*⋯*Cg*^ii^	0.93	2.82	3.641 (2)	147
C21—H21*A*⋯*Cg*^iii^	0.96	2.94	3.546 (3)	122

Symmetry code: (i) 
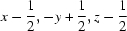
; (ii) 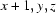; (iii) 
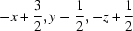
. *Cg* is the centroid of the furanyl ring O1/C1–C4.

**Fig. 1.**
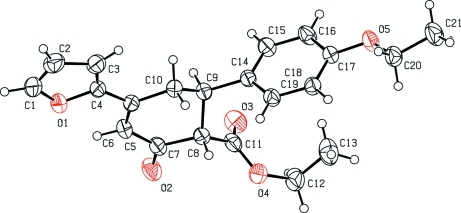
The title molecule showing the atom-labelling scheme. The displacement ellipsoids are drawn at the 40% probability level and the H atoms are shown as small spheres of arbitrary radii.

**Fig. 2.**
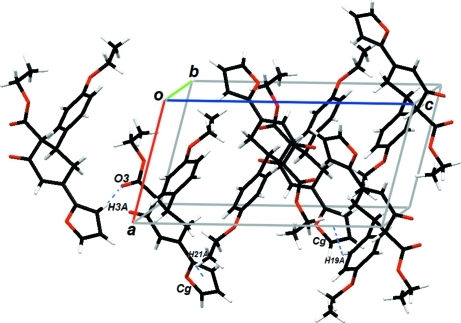
The packing diagram of the title compound, viewed along the the a-axis showing the weak hydrogen-bond and C—H···π electron interactions.

**Fig. 3.**
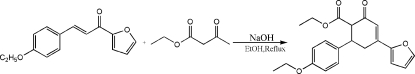
Preparation of the title compound.

## supplementary crystallographic information

### Comment

Cyclohexenones are prepared either from natural sources or entirely *via* synthetic routes. The motive for their preparation is a variety of medical effects. The molecules have anticonvulsant, antimalarial, anti-inflammatory and cardiovascular effects (Eddington *et al.*, 2000). Cyclohexenones are also important intermediates for many biologically active compounds (Padmavathi *et al.*, 2000, 2001). A series of novel compounds has been synthesized, known as cyclohexenoic long chain fatty alcohols, which are used in the treatment of neurological disorders (Luu *et al.*, 2000). A number of their derivatives have fungicidal and antitumor activities (Li *et al.*, 2001).

The title compound (Fig. 1) is a derivative of 1-(furan-2-yl)-3-(4-ethoxyphenyl)prop-2-en-1-one.

Two rings of the title molecule, *i.e.* furan-2-yl [O1\C1 ··· C4] and the cyclohexene [C5 ··· C10], are almost parallel containing 6.77 (11)°. Cyclohexene is approximately perpendicular to the benzene ring [C14 ··· C19], containing 84.79 (05)°. The title molecule has two asymmetric carbon atoms C8 and C9. The respective configurations are *SR* and *RS* within the racemic pair in the structure.

There are weak intermolecular interactions only in the structure that are indicated by geometry, X-H···O and C—H···π-electron ring contacts (Tab. 1). The molecular packing is shown in Fig. 2.

### Experimental

The title compound was synthesized by refluxing ethyl acetoacetate (0.39 g, 0.40 ml, 3 mmol) with 1-(furan-2-yl)-3-(4-ethoxyphenyl)prop-2-en-1-one (3 mmol, 0.726 g) for 2 h in 10–15 ml of ethanol in presence of 0.5 ml 10% NaOH as shown in Fig. 3. The reaction mixture was then poured while having been stirred intensively into 200 ml of ice-cold water. The mixture was kept at room temperature until the reaction product separated as a solid, which was filtered off and recrystallized from ethanol. The grown crystals were prismatic, yellow and with approximate dimensions of 0.4×0.4×0.6 mm. Yield: 70%, m.p.: 388 K.

### Refinement

All the hydrogen atoms could have been discerned in the difference electron density map. Nevertheless, all the H-atoms were placed into idealized positions and refined as riding atoms at constrained distances: C_aryl_—H = 0.93, C_methine_—H = 0.98, C_methylene_—H = 0.97 and C_methyl_—H = 0.96 Å, while *U*_iso_H = 1.5*U*_eq_C_methyl_ or 1.2*U*_eq_C_aryl/methylene/methine_.

### Crystal data

**Table:**

C_21_H_22_O_5_	*F*(000) = 752
*M**_r_* = 354.39	*D*_x_ = 1.313 Mg m^−^^3^
Monoclinic, *P*2_1_/*n*	Melting point: 388 K
Hall symbol: -P 2yn	Mo *K*α radiation, λ = 0.71073 Å
*a* = 7.361 (3) Å	Cell parameters from 86 reflections
*b* = 17.350 (4) Å	θ = 4.6–12.9°
*c* = 14.473 (3) Å	µ = 0.09 mm^−^^1^
β = 104.07 (2)°	*T* = 298 K
*V* = 1792.8 (9) Å^3^	Prism, yellow
*Z* = 4	0.60 × 0.40 × 0.40 mm

### Data collection

**Table:**

Bruker P4 diffractometer	*R*_int_ = 0.021
Radiation source: fine-focus sealed tube	θ_max_ = 30.0°, θ_min_ = 2.4°
graphite	*h* = −10→4
ω scans	*k* = −24→1
8745 measured reflections	*l* = −20→20
5218 independent reflections	3 standard reflections every 97 reflections
3806 reflections with *I* > 2σ(*I*)	intensity decay: 3.4%

### Refinement

**Table:**

Refinement on *F*^2^	Primary atom site location: structure-invariant direct methods
Least-squares matrix: full	Secondary atom site location: difference Fourier map
*R*[*F*^2^ > 2σ(*F*^2^)] = 0.047	Hydrogen site location: difference Fourier map
*wR*(*F*^2^) = 0.135	H-atom parameters constrained
*S* = 1.06	*w* = 1/[σ^2^(*F*_o_^2^) + (0.057*P*)^2^ + 0.3325*P*] where *P* = (*F*_o_^2^ + 2*F*_c_^2^)/3
5218 reflections	(Δ/σ)_max_ < 0.001
237 parameters	Δρ_max_ = 0.27 e Å^−^^3^
0 restraints	Δρ_min_ = −0.17 e Å^−^^3^
86 constraints	

### Special details

**Table:**

Geometry. All e.s.d.'s (except the e.s.d. in the dihedral angle between two l.s. planes) are estimated using the full covariance matrix. The cell e.s.d.'s are taken into account individually in the estimation of e.s.d.'s in distances, angles and torsion angles; correlations between e.s.d.'s in cell parameters are only used when they are defined by crystal symmetry. An approximate (isotropic) treatment of cell e.s.d.'s is used for estimating e.s.d.'s involving l.s. planes.
Refinement. Refinement of *F*^2^ against ALL reflections. The weighted *R*-factor *wR* and goodness of fit *S* are based on *F*^2^, conventional *R*-factors *R* are based on *F*, with *F* set to zero for negative *F*^2^. The threshold expression of *F*^2^ > σ(*F*^2^) is used only for calculating *R*-factors(gt) *etc*. and is not relevant to the choice of reflections for refinement. *R*-factors based on *F*^2^ are statistically about twice as large as those based on *F*, and *R*- factors based on ALL data will be even larger.

### Fractional atomic coordinates and isotropic or equivalent isotropic displacement parameters (Å^2^)

**Table:**

	*x*	*y*	*z*	*U*_iso_*/*U*_eq_	
O1	−0.07980 (14)	0.45302 (6)	0.31575 (9)	0.0611 (3)	
O2	0.43726 (17)	0.45205 (7)	0.60674 (8)	0.0646 (3)	
O3	0.67391 (17)	0.29800 (7)	0.65190 (8)	0.0627 (3)	
O4	0.86811 (14)	0.37514 (6)	0.59822 (8)	0.0571 (3)	
O5	1.03226 (14)	0.08582 (5)	0.40318 (7)	0.0495 (2)	
C1	−0.2189 (2)	0.45660 (10)	0.23520 (15)	0.0697 (5)	
H1A	−0.3227	0.4888	0.2267	0.084*	
C2	−0.1874 (2)	0.40812 (10)	0.16994 (13)	0.0642 (4)	
H2A	−0.2628	0.4004	0.1090	0.077*	
C3	−0.0168 (2)	0.37048 (9)	0.21122 (11)	0.0514 (3)	
H3A	0.0416	0.3328	0.1829	0.062*	
C4	0.04471 (17)	0.39962 (7)	0.29930 (10)	0.0439 (3)	
C5	0.21001 (17)	0.38693 (7)	0.37425 (10)	0.0411 (3)	
C6	0.23954 (19)	0.42500 (8)	0.45737 (11)	0.0488 (3)	
H6A	0.1459	0.4574	0.4679	0.059*	
C7	0.4111 (2)	0.41770 (8)	0.53120 (10)	0.0471 (3)	
C8	0.56299 (18)	0.36875 (7)	0.50669 (9)	0.0422 (3)	
H8A	0.6283	0.4004	0.4688	0.051*	
C9	0.47735 (17)	0.29939 (7)	0.44584 (9)	0.0394 (3)	
H9A	0.3986	0.2718	0.4806	0.047*	
C10	0.34964 (17)	0.33007 (8)	0.35447 (9)	0.0415 (3)	
H10A	0.4249	0.3548	0.3165	0.050*	
H10B	0.2835	0.2874	0.3179	0.050*	
C11	0.7048 (2)	0.34303 (8)	0.59512 (9)	0.0457 (3)	
C12	1.0290 (2)	0.34644 (11)	0.66794 (13)	0.0672 (5)	
H12A	0.9931	0.3332	0.7262	0.081*	
H12B	1.1248	0.3860	0.6827	0.081*	
C13	1.1037 (3)	0.27755 (12)	0.62977 (14)	0.0757 (5)	
H13A	1.2236	0.2645	0.6702	0.114*	
H13B	1.1170	0.2882	0.5667	0.114*	
H13C	1.0189	0.2352	0.6277	0.114*	
C14	0.62510 (16)	0.24380 (7)	0.43058 (8)	0.0373 (2)	
C15	0.6367 (2)	0.17049 (8)	0.46933 (10)	0.0454 (3)	
H15A	0.5501	0.1555	0.5031	0.054*	
C16	0.7726 (2)	0.11937 (8)	0.45920 (10)	0.0482 (3)	
H16A	0.7773	0.0705	0.4861	0.058*	
C17	0.90234 (17)	0.14023 (7)	0.40922 (9)	0.0381 (3)	
C18	0.89386 (18)	0.21308 (7)	0.36978 (10)	0.0420 (3)	
H18A	0.9803	0.2279	0.3358	0.050*	
C19	0.75621 (18)	0.26366 (7)	0.38113 (10)	0.0431 (3)	
H19A	0.7518	0.3126	0.3546	0.052*	
C20	1.1525 (2)	0.10138 (8)	0.34250 (11)	0.0516 (3)	
H20A	1.2328	0.1449	0.3665	0.062*	
H20B	1.0795	0.1137	0.2789	0.062*	
C21	1.2679 (3)	0.03130 (10)	0.33988 (16)	0.0721 (5)	
H21A	1.3438	0.0389	0.2953	0.108*	
H21B	1.1871	−0.0122	0.3206	0.108*	
H21C	1.3471	0.0220	0.4021	0.108*	

### Atomic displacement parameters (Å^2^)

**Table:**

	*U*^11^	*U*^22^	*U*^33^	*U*^12^	*U*^13^	*U*^23^
O1	0.0433 (5)	0.0450 (5)	0.0870 (8)	0.0109 (4)	0.0004 (5)	−0.0034 (5)
O2	0.0676 (7)	0.0628 (7)	0.0603 (6)	0.0169 (5)	0.0094 (5)	−0.0202 (5)
O3	0.0758 (8)	0.0597 (7)	0.0523 (6)	0.0065 (6)	0.0152 (5)	0.0072 (5)
O4	0.0461 (6)	0.0526 (6)	0.0649 (6)	0.0054 (4)	−0.0016 (5)	0.0015 (5)
O5	0.0533 (6)	0.0412 (5)	0.0612 (6)	0.0136 (4)	0.0276 (5)	0.0092 (4)
C1	0.0432 (8)	0.0489 (8)	0.1032 (14)	0.0069 (6)	−0.0088 (8)	0.0089 (9)
C2	0.0507 (8)	0.0561 (9)	0.0740 (10)	−0.0053 (7)	−0.0081 (7)	0.0176 (8)
C3	0.0451 (7)	0.0488 (7)	0.0575 (8)	−0.0019 (6)	0.0068 (6)	0.0090 (6)
C4	0.0351 (6)	0.0335 (6)	0.0620 (8)	0.0017 (5)	0.0095 (5)	0.0077 (5)
C5	0.0351 (6)	0.0344 (6)	0.0540 (7)	0.0007 (5)	0.0110 (5)	0.0047 (5)
C6	0.0429 (7)	0.0422 (7)	0.0612 (8)	0.0107 (6)	0.0123 (6)	−0.0033 (6)
C7	0.0482 (7)	0.0392 (6)	0.0532 (7)	0.0068 (5)	0.0109 (6)	−0.0051 (6)
C8	0.0409 (6)	0.0375 (6)	0.0467 (6)	0.0037 (5)	0.0078 (5)	−0.0007 (5)
C9	0.0384 (6)	0.0371 (6)	0.0435 (6)	0.0038 (5)	0.0119 (5)	−0.0010 (5)
C10	0.0360 (6)	0.0418 (6)	0.0458 (6)	0.0034 (5)	0.0083 (5)	−0.0012 (5)
C11	0.0514 (7)	0.0393 (6)	0.0442 (7)	0.0092 (6)	0.0076 (5)	−0.0061 (5)
C12	0.0538 (9)	0.0695 (10)	0.0660 (10)	0.0137 (8)	−0.0094 (7)	−0.0107 (8)
C13	0.0679 (11)	0.0849 (13)	0.0670 (10)	0.0279 (10)	0.0021 (8)	−0.0060 (9)
C14	0.0368 (6)	0.0366 (6)	0.0381 (6)	0.0038 (5)	0.0081 (5)	−0.0020 (5)
C15	0.0512 (7)	0.0416 (7)	0.0499 (7)	0.0034 (5)	0.0251 (6)	0.0051 (5)
C16	0.0597 (8)	0.0352 (6)	0.0572 (8)	0.0075 (6)	0.0286 (6)	0.0095 (6)
C17	0.0392 (6)	0.0354 (6)	0.0405 (6)	0.0052 (5)	0.0111 (5)	0.0006 (5)
C18	0.0389 (6)	0.0400 (6)	0.0502 (7)	0.0018 (5)	0.0169 (5)	0.0074 (5)
C19	0.0424 (7)	0.0354 (6)	0.0529 (7)	0.0041 (5)	0.0144 (5)	0.0083 (5)
C20	0.0475 (7)	0.0490 (7)	0.0645 (9)	0.0072 (6)	0.0260 (7)	0.0040 (6)
C21	0.0684 (11)	0.0543 (9)	0.1091 (15)	0.0148 (8)	0.0516 (11)	0.0028 (9)

### Geometric parameters (Å, °)

**Table:**

O1—C1	1.353 (2)	C9—H9A	0.9800
O1—C4	1.3642 (16)	C10—H10A	0.9700
O2—C7	1.2186 (17)	C10—H10B	0.9700
O3—C11	1.1949 (18)	C12—C13	1.478 (2)
O4—C11	1.3157 (18)	C12—H12A	0.9700
O4—C12	1.4449 (18)	C12—H12B	0.9700
O5—C17	1.3616 (15)	C13—H13A	0.9600
O5—C20	1.4164 (17)	C13—H13B	0.9600
C1—C2	1.327 (3)	C13—H13C	0.9600
C1—H1A	0.9300	C14—C19	1.3776 (18)
C2—C3	1.413 (2)	C14—C15	1.3842 (18)
C2—H2A	0.9300	C15—C16	1.3710 (19)
C3—C4	1.344 (2)	C15—H15A	0.9300
C3—H3A	0.9300	C16—C17	1.3791 (18)
C4—C5	1.4367 (19)	C16—H16A	0.9300
C5—C6	1.343 (2)	C17—C18	1.3820 (17)
C5—C10	1.5015 (18)	C18—C19	1.3801 (18)
C6—C7	1.448 (2)	C18—H18A	0.9300
C6—H6A	0.9300	C19—H19A	0.9300
C7—C8	1.5133 (19)	C20—C21	1.489 (2)
C8—C11	1.5090 (19)	C20—H20A	0.9700
C8—C9	1.5334 (18)	C20—H20B	0.9700
C8—H8A	0.9800	C21—H21A	0.9600
C9—C14	1.5095 (17)	C21—H21B	0.9600
C9—C10	1.5204 (18)	C21—H21C	0.9600
			
C1—O1—C4	106.29 (13)	O4—C11—C8	109.97 (12)
C11—O4—C12	117.70 (13)	O4—C12—C13	109.51 (13)
C17—O5—C20	117.53 (10)	O4—C12—H12A	109.8
C2—C1—O1	110.96 (14)	C13—C12—H12A	109.8
C2—C1—H1A	124.5	O4—C12—H12B	109.8
O1—C1—H1A	124.5	C13—C12—H12B	109.8
C1—C2—C3	106.45 (15)	H12A—C12—H12B	108.2
C1—C2—H2A	126.8	C12—C13—H13A	109.5
C3—C2—H2A	126.8	C12—C13—H13B	109.5
C4—C3—C2	106.68 (15)	H13A—C13—H13B	109.5
C4—C3—H3A	126.7	C12—C13—H13C	109.5
C2—C3—H3A	126.7	H13A—C13—H13C	109.5
C3—C4—O1	109.60 (12)	H13B—C13—H13C	109.5
C3—C4—C5	133.38 (13)	C19—C14—C15	117.27 (11)
O1—C4—C5	117.00 (13)	C19—C14—C9	122.46 (11)
C6—C5—C4	121.59 (12)	C15—C14—C9	120.24 (11)
C6—C5—C10	121.65 (12)	C16—C15—C14	121.73 (12)
C4—C5—C10	116.75 (12)	C16—C15—H15A	119.1
C5—C6—C7	122.71 (12)	C14—C15—H15A	119.1
C5—C6—H6A	118.6	C15—C16—C17	120.16 (12)
C7—C6—H6A	118.6	C15—C16—H16A	119.9
O2—C7—C6	122.59 (13)	C17—C16—H16A	119.9
O2—C7—C8	121.33 (13)	O5—C17—C16	116.00 (11)
C6—C7—C8	115.95 (12)	O5—C17—C18	124.71 (11)
C11—C8—C7	111.43 (11)	C16—C17—C18	119.28 (11)
C11—C8—C9	110.98 (11)	C19—C18—C17	119.55 (12)
C7—C8—C9	110.55 (11)	C19—C18—H18A	120.2
C11—C8—H8A	107.9	C17—C18—H18A	120.2
C7—C8—H8A	107.9	C14—C19—C18	122.01 (12)
C9—C8—H8A	107.9	C14—C19—H19A	119.0
C14—C9—C10	114.30 (10)	C18—C19—H19A	119.0
C14—C9—C8	112.04 (10)	O5—C20—C21	107.84 (12)
C10—C9—C8	107.80 (10)	O5—C20—H20A	110.1
C14—C9—H9A	107.5	C21—C20—H20A	110.1
C10—C9—H9A	107.5	O5—C20—H20B	110.1
C8—C9—H9A	107.5	C21—C20—H20B	110.1
C5—C10—C9	111.80 (11)	H20A—C20—H20B	108.5
C5—C10—H10A	109.3	C20—C21—H21A	109.5
C9—C10—H10A	109.3	C20—C21—H21B	109.5
C5—C10—H10B	109.3	H21A—C21—H21B	109.5
C9—C10—H10B	109.3	C20—C21—H21C	109.5
H10A—C10—H10B	107.9	H21A—C21—H21C	109.5
O3—C11—O4	125.33 (13)	H21B—C21—H21C	109.5
O3—C11—C8	124.65 (14)		
			
C4—O1—C1—C2	0.19 (19)	C8—C9—C10—C5	−52.86 (14)
O1—C1—C2—C3	0.2 (2)	C12—O4—C11—O3	8.9 (2)
C1—C2—C3—C4	−0.55 (18)	C12—O4—C11—C8	−168.69 (12)
C2—C3—C4—O1	0.68 (16)	C7—C8—C11—O3	68.81 (17)
C2—C3—C4—C5	−177.72 (14)	C9—C8—C11—O3	−54.86 (17)
C1—O1—C4—C3	−0.55 (16)	C7—C8—C11—O4	−113.62 (13)
C1—O1—C4—C5	178.14 (13)	C9—C8—C11—O4	122.71 (12)
C3—C4—C5—C6	179.29 (15)	C11—O4—C12—C13	83.8 (2)
O1—C4—C5—C6	0.99 (19)	C10—C9—C14—C19	58.90 (16)
C3—C4—C5—C10	0.3 (2)	C8—C9—C14—C19	−64.11 (16)
O1—C4—C5—C10	−177.97 (11)	C10—C9—C14—C15	−123.20 (13)
C4—C5—C6—C7	−175.17 (13)	C8—C9—C14—C15	113.80 (14)
C10—C5—C6—C7	3.7 (2)	C19—C14—C15—C16	0.0 (2)
C5—C6—C7—O2	−179.68 (15)	C9—C14—C15—C16	−178.05 (13)
C5—C6—C7—C8	4.3 (2)	C14—C15—C16—C17	−0.1 (2)
O2—C7—C8—C11	23.0 (2)	C20—O5—C17—C16	172.14 (13)
C6—C7—C8—C11	−160.97 (12)	C20—O5—C17—C18	−8.22 (19)
O2—C7—C8—C9	146.87 (14)	C15—C16—C17—O5	179.78 (13)
C6—C7—C8—C9	−37.06 (17)	C15—C16—C17—C18	0.1 (2)
C11—C8—C9—C14	−48.49 (15)	O5—C17—C18—C19	−179.55 (13)
C7—C8—C9—C14	−172.65 (11)	C16—C17—C18—C19	0.1 (2)
C11—C8—C9—C10	−175.09 (11)	C15—C14—C19—C18	0.2 (2)
C7—C8—C9—C10	60.74 (14)	C9—C14—C19—C18	178.21 (12)
C6—C5—C10—C9	21.98 (18)	C17—C18—C19—C14	−0.3 (2)
C4—C5—C10—C9	−159.07 (11)	C17—O5—C20—C21	−174.37 (14)
C14—C9—C10—C5	−178.13 (10)		

### Hydrogen-bond geometry (Å, °)

**Table:**

*D*—H···*A*	*D*—H	H···*A*	*D*···*A*	*D*—H···*A*
C3—H3A···O3^i^	0.93	2.55	3.441 (2)	160
C6—H6A···O1	0.93	2.42	2.761 (2)	102
C19—H19A···Cg^ii^	0.93	2.82	3.641 (2)	147
C21—H21A···Cg^iii^	0.96	2.94	3.546 (3)	122

Symmetry codes: (i) *x*−1/2, −*y*+1/2, *z*−1/2;  (ii) *x*+1, *y*, *z*; (iii) −*x*+3/2, *y*−1/2, −*z*+1/2.
